# Protective Effect of *An-Gong-Niu-Huang Wan* Pre-treatment Against Experimental Cerebral Ischemia Injury via Regulating GSK-3β/HO-1 Pathway

**DOI:** 10.3389/fphar.2021.640297

**Published:** 2021-04-16

**Authors:** Shiqing Zhang, Xiaoli Jiang, Ying Wang, Kaili Lin, Zhang Zhang, Zhu Zhang, Peili Zhu, Man Ling Ng, Shaogang Qu, Stephen Cho Wing Sze, Ken Kin Lam Yung

**Affiliations:** ^1^ Department of Biology, Faculty of Science, Hong Kong Baptist University (HKBU), Hong Kong Special Administrative Region (HKSAR), Kowloon Tong, China; ^2^ HKBU Shenzhen Research Institute and Continuing Education, Shenzhen, China; ^3^ Golden Meditech Center for NeuroRegeneration Sciences, HKBU, HKSAR, Kowloon Tong, China; ^4^ School of Public Health, Guangzhou Medical University, Guangzhou, China; ^5^ Department of Neurology, Nanfang Hospital, Southern Medical University, Guangzhou, China

**Keywords:** an-gong-niu-huang wan, protective effect, cerebral ischemia, GSK-3β/HO-1 pathway, antioxidant properties

## Abstract

*An-Gong-Niu-Huang Wan* (*AGNHW*)*,* a famous formula in traditional Chinese medicine, has been clinically used for centuries for treating cerebral diseases, but the protective effects of pre-treatment with *AGNHW* on cerebral ischemia have not yet been reported. The present study aimed to test such protective effects and elucidate the underlying mechanisms on cerebral ischemia in rats by phenotypic approaches (i.e. including the neurological functional score, cerebral infarct area, neuron apoptosis, and brain oxidative stress status) and target-based approaches (i.e. involving the GSK-3β/HO-1 pathway). AGNHW was administered orally at the doses of 386.26, 772.52, and 1545.04 mg/kg respectively for 7 days to male Sprague-Dawley rats and then cerebral ischemia was induced by middle cerebral artery occlusion (MCAO) for 1.5 h. Pre-treatment with AGNHW significantly ameliorated ischemic damage to the brain in a dose-dependent manner, including reduction of the neurological deficit score and infarct area. AGNHW pre-treatment increased the number of Nissl^+^ cells, NeuN^+^ and DCX^+^ cells, and decreased the number of Tunel^+^ cells. Moreover, AGNHW reversed the up-regulation of ROS and MDA induced by cerebral ischemia. AGNHW pre-treatment increased the expression of p-GSK-3β(Ser9)/GSK-3β (glycogen synthase kinase-3β) ratio and heme oxygenase-1 (HO-1). These results firstly revealed that short-term pre-treatment of AGNHW could significantly protect the rats from injury caused by cerebral ischemia-reperfusion, which support further clinical studies for disease prevention. The *in vivo* protective effect of AGNWH pre-treatment could be associated with its antioxidant properties by the activation of GSK-3β-mediated HO-1 pathway.

## Introduction

Ischemic stroke remains a leading cause of mortality and disability worldwide, with a huge economic burden on the society. Ischemic stroke shows a complex pathophysiological course leading to peroxidation stress, loss of membrane potential, cell depolarization and, eventually, neuronal cell death, involving a variety of distinct molecular and cellular mechanisms of ischemic brain damage (Wan et al., 2020). Although the pharmaceutical industry has been making huge strides in recent years, the lack of effective anti-stroke drugs remains one of the most unmet needs in medicine (Abe et al., 2019). Stroke is a medical emergency but medications for post-stroke treatment are extremely limited in number. A stroke usually causes lasting brain damage, long-term disability, or even death. Therefore, prevention is a more effective strategy than cure in the treatment of stroke.

Traditional Chinese medicine (TCM) with fewer side-effects is often sought to provide alternative medications to prevent ischemic stroke (Liu et al., 2018). The An-Gong-Niu-Huang Wan (AGNHW), a famous formula in TCM, composed of Arisaema Cum Bile, Rhizoma Coptidis, Bombyx Batryticatus, Radix Saposhnikoviae, Borneolum Syntheticum, Bovis Calculus, Moschus, Scorpio, Rhizoma Pinelliae Praeparatum, Radix Trichosanthis, Succinum, Radix Scutellariae, Fructus Amomi, refined honey. AGNHW has been clinically used for treating cerebral diseases for centuries. In Chinese medicine practice, AGNHW with detoxification, resuscitation, and anticonvulsant properties is clinically used for alleviating the clinical syndrome, including loss of consciousness, high fever, recurring seizures, dizziness, vomiting, movement difficulties, neck stiffness, sudden severe headaches, nausea and ataxia, aphasia, and constipation. Recent studies suggested that AGNHW exhibited a therapeutic effect on diseases of the central nervous system (Guo et al., 2014) and injury brought about by experimental cerebral ischemia (Wang et al., 2014; Tsoi et al., 2019). Nevertheless, prevention is better than cure. However, studies about the protective effect of AGNHW pre-treatment on experimental cerebral ischemia injury and its potential mechanism are still lacking.

Oxidative stress caused by reactive oxygen species (ROS) has been considered as one of the underlying mechanisms for inducing neuronal damage by ischemic stroke. Anti-oxidative stress has been considered as a promising therapeutic target and become a hot research topic on ischemic stroke prevention and treatment (Menon et al., 2020). It has been reported that potential anti-oxidant agents, such as carotenoids could be used for stroke prevention (Bahonar et al., 2017). Moreover, pretreatment with Chikusetsu Saponin IVa ameliorated cerebral ischemia reperfusion injury in diabetic mice via inhibition of GSK-3β (increase an inhibitory phosphorylation of GSK-3β at Ser9) (Duan et al., 2016). Paeonol was pre-administered intragastrically once daily for 3 days and with the last administration at 30 min before the operation in the fourth day. Pre-treatment administration of paeonol attenuated cerebral ischemic injury and reduced oxidative stress damage via upregulating expression of HO-1 in the mouse model of MCAO (Zhao et al., 2014). Therefore, the GSK-3β/HO-1 pathway was considered as the important regulator for oxidative stress. Specifically, upregulation of heme oxygenase-1 (HO-1) via GSK-3β deactivation through Ser9 phosphorylation could remarkably suppress the oxidative stress damage (Duan et al., 2019). It has been reported that glycine improved ischemic stroke via activation of the GSK-3β/HO-1 pathway (Chen et al., 2020). These studies suggested that the diminution of oxidant stress by activation of GSK-3β/HO-1 pathway is an effective therapeutic strategy for ischemic stroke prevention and treatment.

We therefore hypothesized that AGNHW pre-treatment could improve the neurological functional score, reduce the cerebral infarct area, inhibit neuronal apoptosis, attenuate the oxidative stress status, and activate the GSK-3β/HO-1 pathway in the MCAO rat model. The aim of this study was to determine whether AGNHW pre-treatment can curb stroke-associated pathological changes and neuronal death in a MCAO rat model by using phenotypic strategies (i.e. including the neurological functional score, cerebral infarct area, neuron apoptosis, brain oxidative stress status) and target-based (i.e. involving the GSK-3β/HO-1 pathway) strategies. Counteraction of oxidation via inhibition of GSK-3β may, therefore, be a promising strategy for prevention of ischemic stroke.

## Materials and Methods

### Animals

The protocol of animal experiment has been officially approved by the Department of Health, the Government of the Hong Kong Special Administrative Region. The animal studies were conducted in accordance with guidelines and security standards of the Committee on the Use of Human and Animal Subjects in Teaching and Research. Male Sprague-Dawley (SD) rats weighing 200–220 g were obtained from the Beijing Viton Lihua Experimental Animal Technology Co., Ltd. (Beijing, China). The animals were kept in a humidity- and climate-controlled environment with food and water *ad libitum* and exposed to a 12–12 h light-dark cycle.

### Administration of AGNHW

AGNHW was kindly provided by Ma Pak Leung Co., Ltd. According to the instruction, the dose of AGNHW used for human was 62.3 mg/kg/day. The equivalent dose for rats was 62.3 × 6.2 = 386.26 mg/kg/day which was used as the low dose of AGNHW (AGNHW-L) in the present study. The intermediate dose of AGNHW (AGNHW-M) and high-dose of AGNHW (AGNHW-H) was twice and quadruple of the clinical equivalent dose, respectively. To monitor the protective effects of AGNHW pre-treatment on experimental cerebral ischemia injury, male SD rats were randomly divided into the following five groups comprising 15 animals per group: Sham operation only, MCAO only, AGNHW-L (386.26 mg/kg) followed by MCAO, AGNHW-M (772.52 mg/kg) followed by MCAO, and AGNHW-H (1545.04 mg/kg) followed by MCAO. AGNHW was dissolved in normal saline solution and then administered intragastrically (i.g.) once daily for 7 consecutive days. Untreated rats were treated with the equivalent volume of physiological saline.

### MCAO Surgical Procedure

The MCAO surgery was performed 2 h after the last administration of AGNHW according to the previous report (Guo et al., 2014). All rats were anesthetized with 3% isoflurane via inhalation. The rats were placed in the supine position on an operating table under sterile conditions. The common carotid artery (CCA), external carotid artery (ECA), and internal carotid artery (ICA) were exposed and ligated on the left side. A monofilament suture with a silicon-coated tip (L3200, Jialing Co. Ltd., China) was inserted into the ECA and advanced through the ICA to the ostium to occlude the middle cerebral artery. Sham control rats were subjected to a similar surgical operation without occlusion. After 1.5 h of ischemia, the monofilament suture was withdrawn to permit reperfusion. The reperfusion process was continued for 24 h and then the neurological status was scored. All animals were then transcardially perfused with PBS under anesthesia to collect brain tissues for further experiments respectively: the 5 brains were used for measurements of the infarct sizes; the 5 brains were stored in 10% neutral formaldehyde solution and used for histopathological investigation; the last 5 brains were used for the measurement of reactive oxygen species (ROS), malondialdehyde (MDA) and the activation of GSK-3β/HO-1 pathway.

### Functional Assessment

The neurological status of all rats (*n* = 15) was scored at 24 h after reperfusion. The degree of neurological function was assessed by the Zea-Longa score with a five-point scale: grade 0 corresponds to symptoms without neurological impairment (normal); grade 1 corresponds to inextensibility of the left forepaw when lifting the rats' tail(mild); grade 2 corresponds to circling to the left side while walking (moderate); grade 3 corresponds to walking difficulty and leaning to the left (severe); and grade 4 corresponds to inability to walk spontaneously (very severe) (Guo et al., 2019).

### Measurement of Infarct Area

Rats were sacrificed after 24 h of reperfusion under anesthesia and their brains were excised for estimation of the infarct area (*n* = 5). The brains were sectioned into 2 mm slices with a rat brain matrix (RWD Life Science, Shenzhen, China) and placed in a 2% 2,3,5-triphenyltetrazoliumchloride (TTC, Sigma-Aldrich, MO, United States) solution for 20 min in the dark. Infarct size was quantified by measuring the white infarcted area and red-purple non-infarcted area using the ImageJ software. The ratio of the infarct area to the total area was calculated.

### Histologic Procedures and Nissl Staining

Nissl staining was performed as previously reported (Lin et al., 2021). Twenty four hours after reperfusion, the rats (*n* = 5) were anesthetized and perfused transcardially with cold saline and 4% paraformaldehyde (PFA, Sigma-Aldrich, United States). The brains were rapidly removed, immersed in the fixative for 48 h and then embedded in paraffin and micro-sectioned into coronal slices of 5 μm thickness. The coronal brain sections were stained using 0.5% cresyl violet acetate (Beyotime, Beijing, China). The severity of neural damage was monitored by counting the number of normal neurons in infarct tissues under a Pannoramic DESK scanner from 3D-HISTECH (Hungary). The cells that contained the Nissl stain in the cytoplasm, loose chromatin and prominent nucleoli were considered to be normal neurons. Stained cells in five lesioned regions of the ischemic cortex were counted randomly.

### Immunofluorescent and TUNEL Staining

Immunofluorescent staining with the appropriate primary antibodies (anti-NeuN and anti-DCX) and TUNEL staining with an in-situ Cell Death Detecting kit (Roche Diagnostics GmbH, Penzberg, Germany) were performed as previously described (Peng et al., 2019). Briefly, the brain sections were incubated with primary antibodies at 4°C overnight. After washing with PBS for three times, the sections were incubated with the appropriate secondary antibodies conjugated to fluorescein isothiocyanate. DAPI (1 μg/ml) was then used to counterstain the nuclei. For TUNEL staining, the brain sections were incubated with the TUNEL reaction mixture in the chamber at room temperature, and after washing with PBS, the slices were stained with DAPI. Optical and metric analysis of the stained tissues was performed with a Pannoramic DESK scanner from 3D-HISTECH (Hungary). All of the stained cells in the image were counted, including NeuN^+^, Sox2^+^ and TUNEL^+^ cells. Stained cells in five lesioned regions of the ischemic cortex were counted randomly.

### Measurements of ROS and MDA

Twenty four hours after reperfusion, infarct tissues from the rats (*n* = 5) were harvested and homogenized before use for measurements of ROS and MDA and for the Western blotting assay. The homogenate was centrifuged at 2,000 × *g* at 4°C for 15 min. The supernatant was evaluated to determine the content of ROS and MDA using assay kits (ROS, cat. no. E004-1-1; MDA, A003-1-1; Nanjing Jiancheng Bioengineering Institute, Nanjing, China) according to the manufacturer's protocol and monitored by a spectrophotometer (UV-2600; Shimadzu Corporation, Kyoto, Japan).

### Western Blotting

Proteins were then extracted from the homogenate on ice using a protein extraction reagent supplemented with a protease inhibitor (Novagen, Madison, WI, United States). Equal amounts of protein samples were separated in 10% SDS-polyacrylamide gel electrophoresis and then transferred to a PVDF membrane (Bio-Rad Laboratories). After blocking in 5% defatted milk for 1 h, the membranes were subsequently incubated with appropriate primary antibodies at 4°C overnight: p-GSK-3β(Ser9), GSK-3β, and HO-1. The membranes were then incubated with the appropriate secondary antibodies for 1 h. The β-actin protein was used as internal loading control. Images were captured using a ChemiDoc Touch imaging system (Bio-Rad Laboratories) and intensities of the protein bands were analyzed using ImageJ (NIH, United States).

### Statistical Analyses

The quantitative results are presented as the means ± standard deviation (SD). Data were analyzed statistically by using one-way analysis of variance, and difference between groups was considered statistically significant at *p* < 0.05 or smaller. Image analysis and cell counting were performed in ImageJ. All graphs were produced using the GraphPad Prism 5.0 software (San Diego, CA, United States).

## Results

### Protective Effect of Pre-treatment With AGNHW on Injury Induced by Experimental Cerebral Ischemia

**FIGURE 1:**
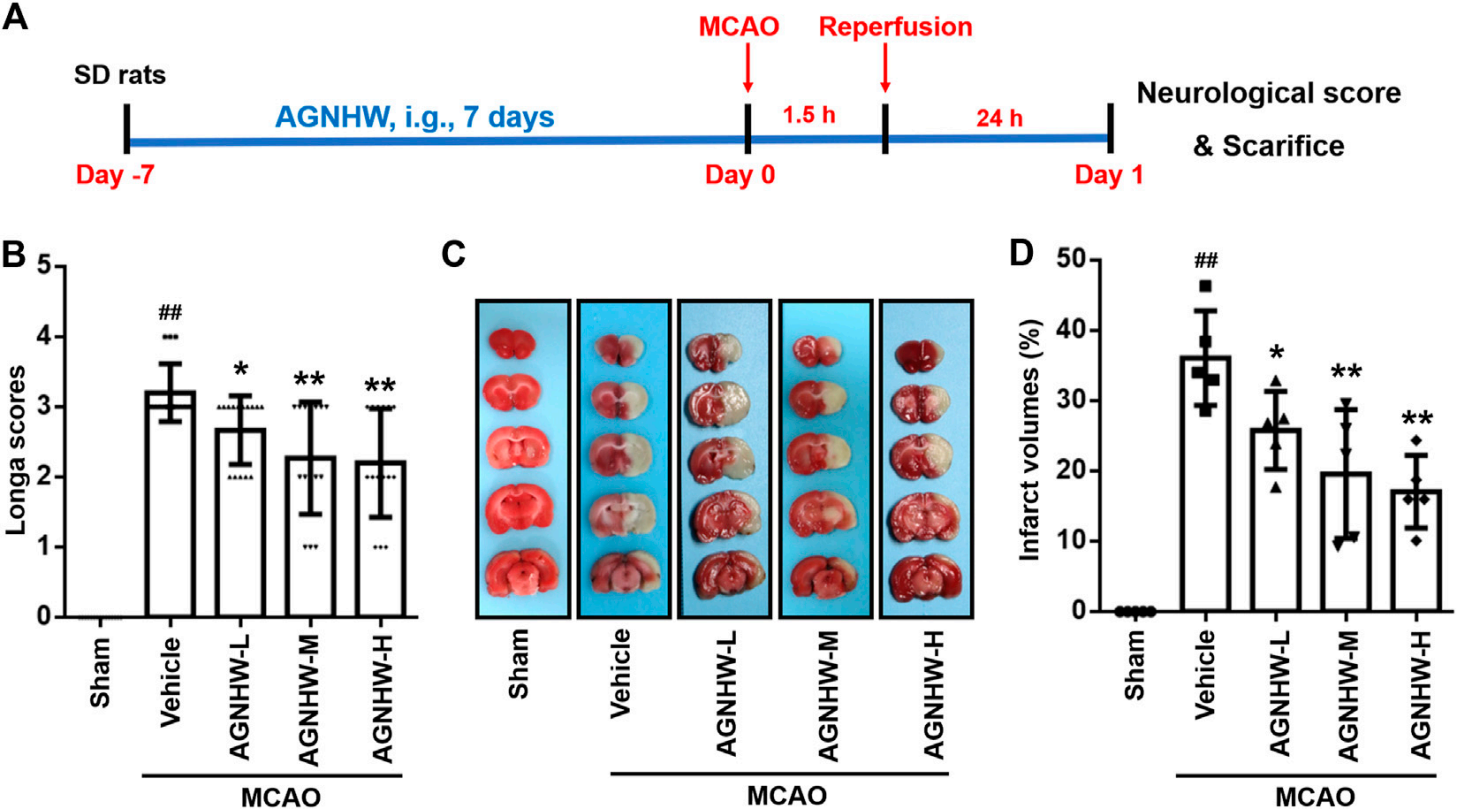
AGNHW pre-treatment ameliorated neurological deficit and decreased infarct size in MCAO rat model **(A)** Illustration of experimental schedule. **(B)** The degree of neurological function was assessed by Zea long’s scoring on a five-point scale 24 h after reperfusion (*n* = 15) **(C,D)** Infarct size was measured by TTC staining 24 h after reperfusion (*n* = 5). Representative image **(C)** and statistical analysis of results of TTC staining **(D)**. Data are means ± S.D. ^##^ *p* < 0.01, compared with the sham group; *p < 0.05 and **p < 0.01, compared with the vehicle group.

The protective effect of AGNHW pre-treatment was monitored by amelioration of neurological deficits and reduction of infarct area. Neurological deficit was evaluated by Zea long’s scoring at 24 h after reperfusion. The neurological scores were significantly higher in the MCAO group than the sham group. Compared with the MCAO group, the neurological scores were markedly reduced in the AGNHW groups in a dose-dependent manner. Statistically significant difference was observed 24 h after reperfusion between the sham group and groups treated with the various doses of AGNHW (386.26, 772.52 and 1545.04 mg/kg) (Figures 1A,B). Additionally, the infarct areas in the MCAO rats were measured by TTC staining. As shown in Figures 1C,D no infarct was observed in the sham group, while rats in the MCAO group showed extensive lesions in both striatum and lateral cortex 24 h after reperfusion. Compared with the MCAO group, the infarct areas in the MCAO rats pre-treated with the various doses of AGNHW (386.26, 772.52 and 1545.04 mg/kg) were reduced significantly in a dose-dependent manner.

### AGNHW Pre-treatment Mediated Neuroprotection

**FIGURE 2:**
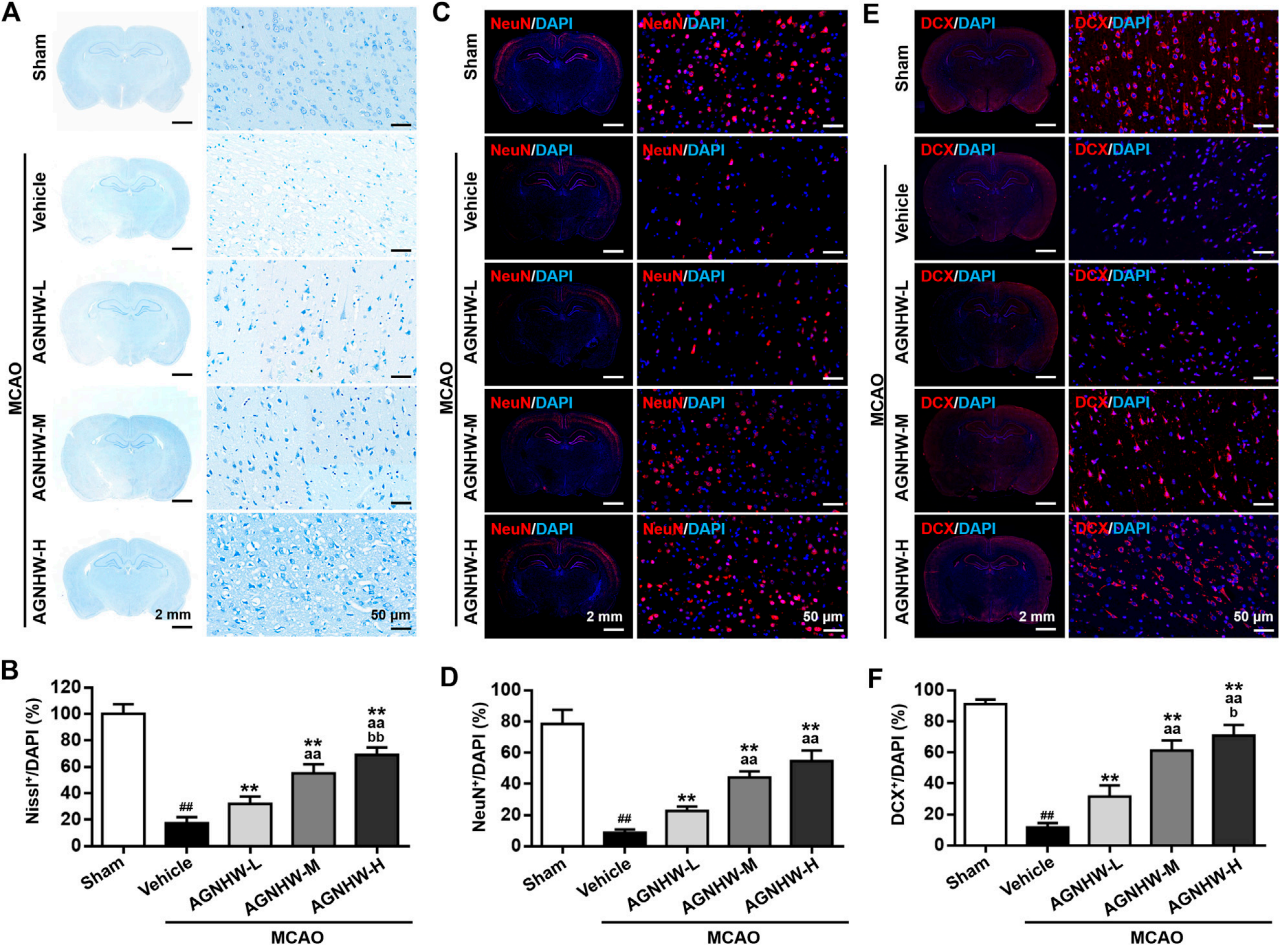
AGNHW pre-treatment restored neuronal loss in MCAO model **(A,B)** Representative images (**A**) and statistical analysis results (**B**) of Nissl staining in the infarct area of the ischemic cortex in MCAO rats with or without pre-treatment with AGNHW **(C,D)** Representative images (**C**) and statistical analysis results (**D**) of immunofluorescent staining to visualize NeuN in the infarct area of the ischemic cortex in MCAO rats with or without pre-treatment with AGNHW **(E,F)** Representative images (**E**) and statistical analysis results (**F**) of immunofluorescent staining to visualize DCX in the infarct area of the ischemic cortex in MCAO rats with or without pre-treatment with AGNHW. *N* = 5 rats per group. Data are means ± S.D. ^##^ *p* < 0.01, compared with the sham group; **p < 0.01, compared with the vehicle group; ^aa^ *P*<0.01, compared with AGNHW-L group; ^b^ *P*<0.05 and ^bb^ *P*<0.01, compared with the AGNHW-M group.

Nissl staining and immunofluorescent staining were performed to visualize the expression of NeuN and DCX in ischemic penumbra of cortex after 24 h of reperfusion. Regarding Nissl staining, the percentage of Nissl-positive cells in the ischemic cortex in the MCAO group was significantly decreased compared with the Sham group. After pre-treatment with AGNHW, the percentage of Nissl-positive cells was significantly increased in a dose-dependent manner (Figures 2A,B). Additionally, most of the Nissl-positive cells in MCAO group underwent a reduction in size with an increased intercellular space, which was ameliorated after AGNHW pre-treatment (Figure 2A). The same tendency was also observed in immunofluorescent staining. Specifically, the percentages of NeuN-positive and DCX-positive cells were significantly reduced in the MCAO group relative to the Sham group, which was significantly ameliorated after AGNHW pre-treatment in dose-dependent manner (Figures 2C,D). These results indicated the pre-treatment with AGNHW significantly restored the neural loss in injury associated with experimental cerebral ischemia.

### AGNHW Pre-treatment Inhibited Cellular Apoptosis

**FIGURE 3:**
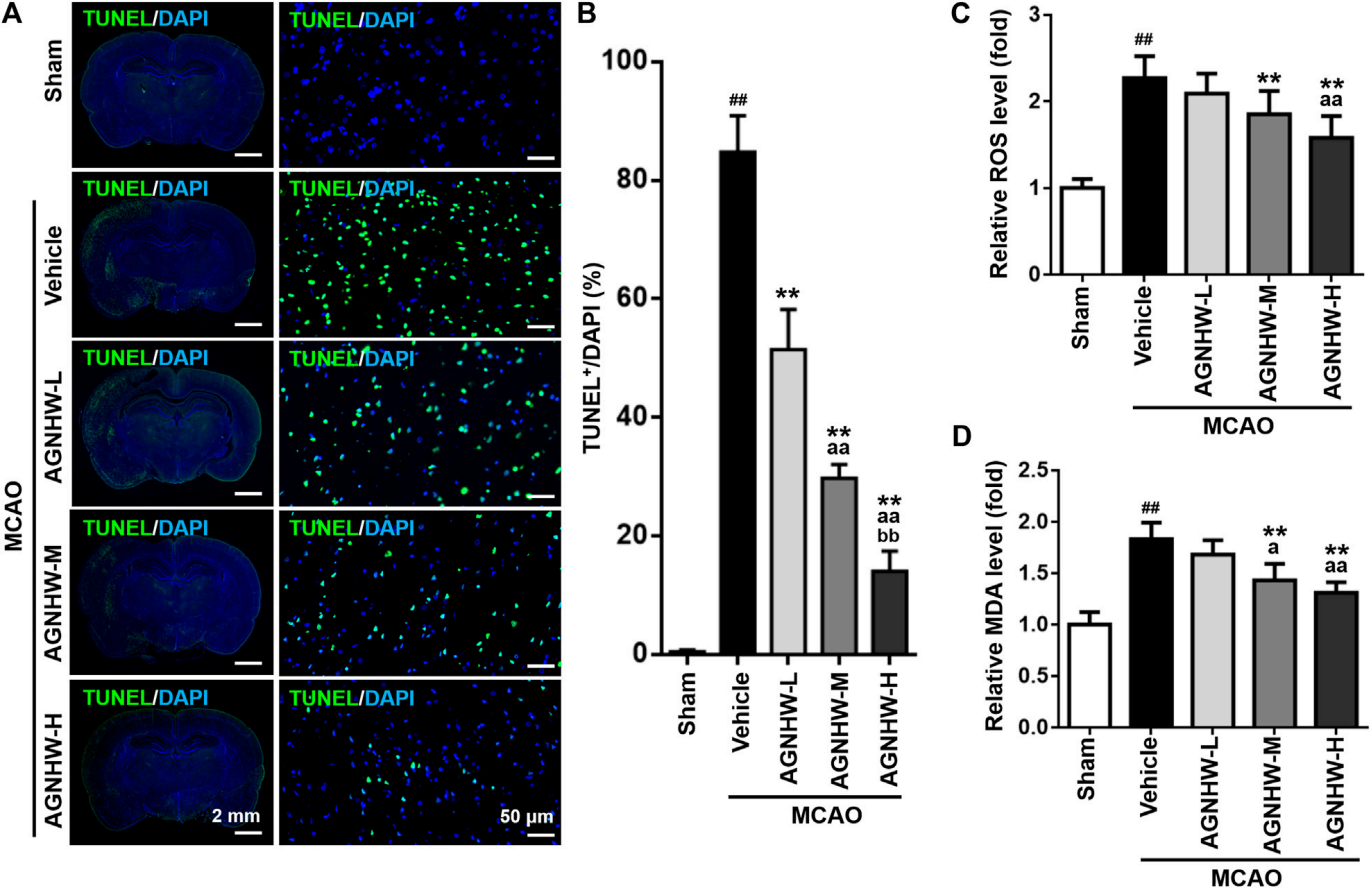
Pre-treatment with AGNHW suppressed cell apoptosis and oxidative stress damage in MCAO model **(A,B)** Representative images (**A**) and results of statistical analysis (**B**) of TUNEL staining in the infarct area of the ischemic cortex in MCAO rats with or without pre-treatment with AGNHW **(C, D)** The status of oxidative stress was monitored by cerebral ROS (**C**) and MDA (**D**) levels, analyzed by appropriate assay kits. *N* = 5 rats per group. Data are means ± S.D. ^##^ *p* < 0.01, compared with sham group; **p < 0.01, compared with vehicle group; ^a^ *P* < 0.05 and ^aa^ *P*<0.01, compared with AGNHW-L group; ^bb^ *P*<0.01, compared with AGNHW-M group.

Cellular apoptosis in the ischemic cortex was analyzed by TUNEL staining 24 h after reperfusion. There was a marked increase in the number of TUNEL-positive cells in the ischemic cortex in the MCAO group compared with the sham group. In contrast, the groups treated with AGNHW demonstrated a significant dose-dependent reduction TUNEL staining compared with the MCAO group. Specifically, the percentage of TUNEL-positive cells was significantly reduced in the AGNHW-H group compared with the AGNHW-M group which was in turn significantly lower than that of the AGNHW-L group (Figures 3A,B). These results strongly suggested that cell apoptosis was attenuated by AGNHW pre-treatment.

### AGNHW Pre-treatment Mitigated Oxidative Stress–Elicited Damage

To investigate the effect of pre-treatment with AGNHW on the damage brought about by oxidative stress in MCAO rats, the levels of ROS and MDA were determined. The level of MDA is an index of lipid peroxidation as the biomarker for oxidative stress. Both ROS and MDA levels were significantly higher in infarct tissues of rats in the MCAO group than those in the sham group (Figures 3C,D). AGNHW pre-treatment exerted a significant dose-dependent effect on reducing the ROS and MDA levels in infarct tissue, indicating a protective effect of AGNHW on oxidative stress–elicited damage.

### AGNHW Pre-treatment Enhanced the Activation of GSK-3β/HO-1 Pathway

**FIGURE 4:**
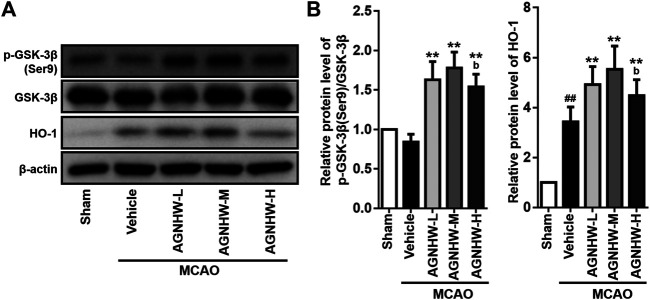
Pre-treatment with AGNHW enhanced activation of GSK-3β/HO-1 pathway. Western blot analysis **(A)** and the normalized protein levels **(B)** of the indicated protein markers of the GSK-3β/HO-1 pathway in the infarct area of the ischemic cortex in MCAO rats with or without AGNHW pre-treatment. AGNHW pre-treatment significantly increased the ratio of *p*-GSK-3β(Ser9)/GSK-3β and the expression of HO-1 in the infarct area of MACAO rats. Full length blots are shown in Supplementary Figure S1. *N* = 4 rats per group. Data are means ± S.D. ^##^ *p* < 0.01, compared with sham group; **p < 0.01, compared with vehicle group; ^b^ *P*<0.05, compared with AGNHW-M group.

The GSK-3β/HO-1 pathway was monitored by Western blot assay. As shown in Figure 1, cerebral ischemia for 1.5 h followed by 24 h of reperfusion downregulated the ratio of p-GSK-3β(Ser9)/GSK-3β, and markedly upregulated HO-1 expression in the rat infarct tissue. Compared with the MCAO group, AGNHW pre-treatment significantly up-regulated the expression ratio of p-GSK-3β(Ser9)/GSK-3β, indicating the inactivation of GSK-3β, and the expression level of HO-1 at 24 h after reperfusion with the highest peak appeared in the AGNHW-M (772.52 mg/kg) group (Figure 4). These results indicated that AGNHW pre-treatment significantly activated the GSK-3β/HO-1 pathway in MCAO rats.

## Discussion

Stroke is the third leading cause of death in China ranking just after malignant tumours and heart disease and the top 10 causes of death worldwide (Wang et al., 2020). To date, the vast majority of pharmacological treatments fails to effectively ameliorate the sequelae of ischemic stroke. A promising strategy underway utilizes tissue plasminogen activator (tPA) as a thrombolytic therapeutic. However, significant side effects limit its use to only a small number of patients. Furthermore, the therapeutic time window of tPA is extremely limited and requires more specialized equipment for successful implementation (Tseng et al., 2020). Consequently, the bulk of stroke patients does not receive any specific pharmacological therapy, ensuing in high mortality and disability rates. Hence, it is imperative to devise potential neuroprotective or therapeutic strategies to forestall or treat ischemic stroke.

Prevention is a better strategy than cure in the treatment of stroke. In this study, we firstly demonstrated that pre-treatment with AGNHW for 7 days prior to MCAO significantly improved the neurological functional score and reduced the cerebral infarct area in the MCAO rat model in a dose-dependent manner. Additionally, in the present study, the human equivalent dose of AGNHW (low-dose group, 386.26 mg/kg/day) displayed a pronounced protective effect on the rat model of MCAO. Mechanistic studies proved that pre-treatment with AGNHW could increase an inhibitory phosphorylation of GSK-3β at Ser 9 without affecting the expression level of total GSK-3β protein and subsequently promote the protein expression of its downstream target HO-1. A number of studies have indicated that inhibition of GSK-3β ameliorated cerebral ischemia injury (Chuang et al., 2011; Venna et al., 2015; Pang et al., 2016; Wang et al., 2017; Gao et al., 2020; Wen et al., 2020), consistent with our findings. Therefore, the effect of AGNHW pre-treatment might be attributed to its protective action on neurons against oxidative stress damage via activation of the GSK-3β-mediated HO-1 pathway.

The antioxidant response element (ARE)–mediated antioxidant pathway plays an important role in maintaining the redox status. Heme oxygenase-1 (HO-1) has been reported to have the most AREs in its promoter, making it a promising therapeutic target against brain injury in cerebral infarction (Bereczki et al., 2018). GSK-3β has emerged as the integration point and is pivotal in switching off the self-protective antioxidant stress response, thus dictating the magnitude and duration of the HO-1 antioxidant response. It has been proven in many studies that inhibition of GSK-3β effectively upregulates HO-1 protein expression (Jiang et al., 2015; Yan et al., 2020). As a result, enhancement of HO-1 via GSK-3β inactivation (GSK-3β/HO-1 pathway) plays a crucial role in improving ischemic stroke (Chen et al., 2020). In summary, HO-1 up-regulation is an adaptive response to oxidative stress in the body. The relationship between GSK-3β and HO-1 pathway in the process of cerebral ischemia is shown as follows: significant amounts of oxidants are generated during cerebral ischemia/reperfusion, and oxidative stress causes brain damage after stroke.

In this study, we found that the expression level of HO-1 was dramatically increased by 1.5 h of cerebral ischemia followed by 24 h of reperfusion, consistent with the previous report that increased HO-1 activity begin immediately after ischemia and continue for 24 h (Shah et al., 2019). Further overexpression of HO-1 by AGNHW pre-treatment reduced oxidative stress in a rat model of ischemic stroke. Therefore, inactivation of GSK-3β could serve as a novel therapeutic target for the protection of stroke and this needs further studies.

Some studies revealed that the inhibition of GSK3β phosphorylation at Tyr 216 deactivated GSK-3β, resulting in a benefit for cerebral ischemia (Chen et al., 2016; Wang et al., 2019). On the other hand, the regulation of GSK-3β usually also depends on phosphorylation within the amino-terminal domain of GSK-3β (Ser 9), resulting in inactivation of GSK-3β. In our present study, we demonstrated that pre-treatment with AGNHW significantly inhibited GSK-3β activation by enhancing GSK-3β phosphorylation at Ser9. A number of studies have indicated that inhibition of GSK-3β contributed to an amelioration of cerebral ischemia injury (Chuang et al., 2011; Venna et al., 2015; Pang et al., 2016; Wang et al., 2017; Gao et al., 2020; Wen et al., 2020), which is consistent with our findings. In a further study, we will test another hypothesis whether AGNHW pre-treatment significantly inhibits GSK-3β phosphorylation at Tyr 216 for cerebral ischemia, before performing the clinical study. Some studies have shown that inhibition of GSK3β phosphorylation is beneficial to cerebral ischemia, the differences between this and the paragraph above are shown as follows: the regulation of GSK-3β usually depends on phosphorylation at its Ser 9 and/or Tyr 216.

It was reported that *Rhizoma Coptidis,* an important traditional Chinese herb in AGHNW, was used for aging-related diseases treatment via antioxidative effect by HO-1 activation (Xu et al., 2017). It was also found that *baicalin* and *baicalein* are flavonoids extracted from *Scutellaria baicalensis*, another traditional Chinese herb in AGHNW, could be effective in the treatment of cerebral ischemia via amelioration of oxidation stress damage (Liang et al., 2017). However, until now, it is not clear which ingredients in AGNHW are responsible for the AGNHW-induced protective effect on MCAO via GSK-3β-mediated HO-1 pathway activation, which need further investigation.

Besides traditional Chinese herbs, there are some heavy metal components, such as arsenic and mercury, in AGNHW, leading to a safety risk of AGNHW. This explains the prohibition of its sale in the US and European markets. In the present study, the commercially available product AGNHW without cinnabar and realgar has been used in this study due to the safety concerns. No significant toxicity was detected during the administration of AGNHW. Additionally, it was reported that the expression of biomarker of metal toxicity metallothionein-1 was not altered by AGNHW (used at 6-fold of the clinical dosage) which was orally administered daily for six weeks in the mouse model (Lu et al., 2011). Published clinical evidence from 1974 to 2015 indicates that the risk of adverse drug reactions and adverse events from AGNHW administration was very low (Zhao et al., 2015). Although some studies also suggested that subchronic use of cinnabar or realgar-containing herbal products could elicit mild renal injury (Wang et al., 2015; Luo et al., 2017), it has been reported it is not very likely that usage of AGNHW at a regular dose for a short duration would induce the accumulation of arsenic and mercury and influence liver and kidney functions (Tsoi et al., 2019). Thus, we conclude that AGNHW is relatively safe when used in short term for ischemic stroke, and it is strongly desirable to have well-designed experiments to systematically study the toxicological effects of prolonged usage of AGNHW (with cinnabar and realgar).

In the present study, AGNHW brought about a dose-dependent decline in the number of TUNEL-positive apoptotic cells in the ischemic brain cortex. This is in keeping with the earlier demonstration that AGNHW administration protected rats from cerebral ischemia–reperfusion injury induced by MCAO. The reduction in the percentages of Nissl-positive, NeuN-positive and DCX-positive cells caused by MCAO was alleviated by AGNHW. AGNHW treatment up-regulated Bcl-2 expression (Wang et al., 2014; Tsoi et al., 2019) and down-regulated the expression of Bax (Wang et al., 2014; Tsoi et al., 2019), caspase-3 (Wang et al., 2014), p47_phox_, inducible nitric oxide synthase, and 3-nitrotyrosine in the ischemic brains (Tsoi et al., 2019). In the present investigation, AGNHW pre-treatment exerted a significant dose-dependent effect on reducing the levels of reactive oxygen species and malondialdehyde in infarct tissue in the ischemic brain cortex, indicating a protective effect of AGNHW on oxidative stress–elicited damage. Tsoi et al. showed that AGNHW exerted neuroprotective effects and minimized cerebral ischemia–reperfusion injury e.g. alleviated oxidative and nitrative (peroxynitrite) stress-mediated matrix metalloproteinase activation and protecting tight junction proteins ZO-1 and claudin-5, which are important components in maintaining the blood-brain barrier. (Tsoi et al., 2019). Malondialdehyde level was elevated and total antioxidant power was diminished in stroke patients compared with control subjects (Menon et al., 2020). AGNHW suppressed lipopolysaccharide-induced reduction of dopamine uptake; inhibited formation of intracellular reactive oxygen species and the mRNA expression of cyclooxygenase-2, inducible nitric oxide synthase, interleukin-1beta and tumor necrosis factor-alpha, and the release of interleukin-1beta, tumor necrosis factor-alpha, and prostaglandin E2 and the protein level of inducible nitric oxide synthase (Li et al., 2018). Data from the various research laboratories all indicated a beneficial action of AGNHW on the ischemic brain.

In conclusion, the present report is the first which demonstrates that pre-treatment of a rat model of ischemic stroke with AGNHW for 7 consecutive days effectively curtailed the injury done in the ischemic brain via activation of GSK-3β-mediated HO-1 pathway. The GSK-3β inhibitor TWS119 given intraperitoneally exhibited a mitigating effect on damage induced by MCAO (Wang et al., 2017). As a neuroprotective agent HO-1 minimizes the damage caused by cerebral ischemia (Song et al., 2019). Our findings strongly suggested that AGNHW pre-treatment has the potential to exert a protective effect *in vivo* against ischemic stroke or other relative neuronal diseases, and inactivation of GSK-3β could serve as a novel therapeutic target for the protection against stroke, which support further clinical studies for disease prevention.

## Data Availability

The raw data supporting the conclusions of this article will be made available by the authors, without undue reservation.
