# Supplementary material for: Protective Effect of An-Gong-Niu-Huang Wan Pre-treatment Against Experimental Cerebral Ischemia Injury via Regulating GSK-3β/HO-1 Pathway
Source: Front Pharmacol. 2021 Apr 16;12:640297. doi: 10.3389/fphar.2021.640297 (PMC8085595; doi:10.3389/fphar.2021.640297)
Supplement: Supplementary file 1 [file datasheet1.docx]

***Supplementary Information***

**Preventive Effect of *An-Gong-Niu-Huang Wan* against Experimental Cerebral Ischemia Injury via Regulating GSK-3β/HO-1 Pathway**

Shiqing Zhang^1,2,3, #^, Xiaoli Jiang^1,3, #^, Ying Wang^1,3^, Kaili Lin^4,1,3^, Zhang Zhang^1,3^, Zhang Zhu^1,2,3^, Peili Zhu^1,2,3^, Man Ling Ng^1,3^, Shaogang Qu^5^, Stephen Cho Wing Sze^1,2,3, **^, Ken Kin Lam Yung^1,2,3, *^

^1^ Department of Biology, Faculty of Science, Hong Kong Baptist University (HKBU), Kowloon Tong, Hong Kong Special Administrative Region (HKSAR), China

^2^ HKBU Shenzhen Research Institute and Continuing Education, Shenzhen, China

^3^ Golden Meditech Center for NeuroRegeneration Sciences, HKBU, Kowloon Tong, HKSAR, China.

^4^ School of Public Health, Guangzhou Medical University, Guangzhou, China

^5^ Department of Neurology, Nanfang Hospital, Southern Medical University, Guangzhou, Guangdong, China.

*Corresponding author: Ken Kin Lam Yung,

Email: [kklyung@hkbu.edu.hk](mailto:kklyung@hkbu.edu.hk); Tel: +852-34117060

**Co-corresponding author: Stephen Cho Wing Sze,

Email: scwsze@hkbu.edu.hk; Tel: +852-3411 2318

^#^These two authors contributed equally to this work.


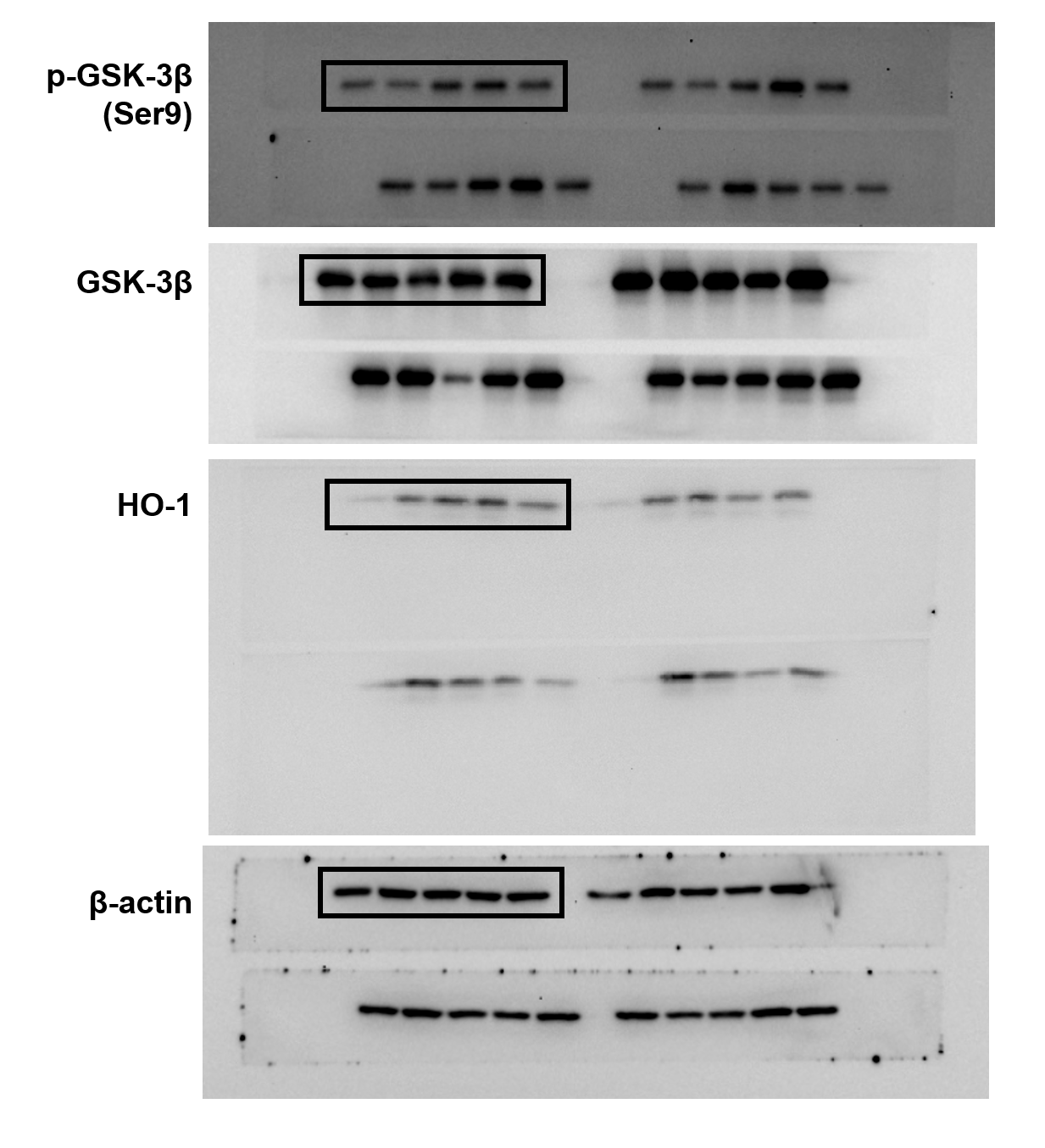


Supplementary Figure 1. Full length blots of Figure 4A (n=4). Western blot analysis of the indicated protein markers of the GSK-3β/HO-1 pathway in the infarct area of the ischemic cortex in MCAO rats with or without pre-treatment with AGNHW. The highlighted bands are represented inserts, as shown in Figure 4A.

*Western blotting Methodology:* Proteins were then extracted from the homogenate on ice using a protein extraction reagent supplemented with a protease inhibitor (Novagen, Madison, WI, USA). Equal amounts of protein samples were separated on a 10% SDS-polyacrylamide gel electrophoresis and then transferred to a PVDF membrane (Bio-Rad Laboratories). After blocking in 5% defatted milk for 1 h, the membranes were split according to the molecular weight of protein of interest, then the split membranes were subsequently incubated with their appropriate primary antibodies at 4 °C overnight: p-GSK-3β(Ser9), GSK-3β, and HO-1. The membranes were then incubated with the appropriate secondary antibodies for 1 h. The β-actin protein was used as internal loading control. Images were captured using a ChemiDoc Touch imaging system (Bio-Rad Laboratories) and intensities of the protein bands were analyzed using ImageJ (NIH, USA).
